# Crude Venom from Sea Anemone *Macrodactyla doreensis* Suppresses Glioblastoma via the p53 Pathway

**DOI:** 10.3390/md24030092

**Published:** 2026-02-26

**Authors:** Limin Lin, Meiling Huang, Wanting Yang, Ziqiang Hua, Zhen Chen, Panmin He, Kailin Mao, Shuanghuai Cheng, Linlin Ma, Shuaiying Cui, Bo Yi, Bingmiao Gao

**Affiliations:** 1 Engineering Research Center of Tropical Medicine Innovation and Transformation of Ministry of Education, Hainan Key Laboratory for Research and Development of Tropical Herbs, International Joint Research Center of Human-Machine Intelligent Collaborative for Tumor Precision Diagnosis and Treatment of Hainan Province, School of Pharmacy, Hainan Medical University, Haikou 571199, Chinamaokailin@muhn.edu.cn (K.M.);; 2 Institute for Biomedicine and Glycomics, School of Environment and Science, Griffith University, Nathan, QLD 4111, Australia; 3 Department of Medicine, Section of Hematology-Medical Oncology, Boston University Chobanian & Avedisian School of Medicine, Boston, MA 02118, USA; 4 Department of Pharmacy, 928th Hospital of PLA Joint Logistics Support Force, Haikou 571199, China

**Keywords:** *Macrodactyla doreensis*, sea anemones, antiglioma activity, p53 signaling pathway, proteomics

## Abstract

Glioblastoma is a highly invasive primary brain tumor with a poor prognosis, highlighting the need for new therapeutic strategies. Toxins derived from *Macrodactyla doreensis* have attracted attention for their potential anticancer activity. This study evaluated the anticancer and cytotoxic effects of *M. doreensis* crude venom on two commonly used glioblastoma cell lines (U251 and LN229), which mirror the phenotype of primary tumors. Cell viability and proliferation were assessed using the CCK-8 assay and colony formation assay, while cell migration and invasion capabilities were detected via wound healing assay and Transwell assay. Annexin V/PI staining and PI-based cell cycle analysis indicated that the crude venom significantly induced cell apoptosis and caused S-phase arrest. Proteomic analysis combined with GO and KEGG enrichment analyses as well as bioinformatics approaches showed that *M. doreensis* crude venom inhibits glioblastoma cell proliferation by downregulating the expression of CDK2, RRM2, and CHEK1, thereby hindering cell cycle progression and regulating the p53 signaling pathway. Notably, the downregulation of these key glioblastoma-related target genes was validated by qPCR. In addition, network pharmacology analysis indicated that several peptide families present in the sea anemone crude venom, including ShK peptides, inhibitor cystine knot (ICK) peptides, and EGF-like peptides, exhibit notable antitumor potential. Combined with AlphaFold2-based structural modeling and molecular docking, these analyses further elucidated the potential molecular mechanisms underlying their interactions with key targets, such as MD-381 with RRM2, MD-322 with CDK2, and MD-429 with CHEK1. Collectively, these findings highlight the therapeutic potential of *M. doreensis* crude venom and lay a foundation for the subsequent isolation of novel peptides and their further development in glioblastoma treatment.

## 1. Introduction

Sea anemones, belonging to the phylum Cnidaria, produce venoms that consist of complex mixtures of diverse bioactive compounds, including peptides, proteins, protease inhibitors, and biogenic amines. These components play essential roles in prey capture and defense mechanisms [1]. The nematocysts located on the tentacles of sea anemones serve as their primary offensive and defensive apparatus. Upon mechanical or chemical stimulation, the nematocysts rapidly discharge venom to facilitate prey capture or deter predators. Beyond their ecological significance, sea anemone toxins have attracted sustained scientific interest due to their high specificity in modulating ion channels, signal transduction pathways, and cellular metabolism, making them promising scaffolds for natural product-based drug discovery and development [2].

Our research group previously established comprehensive peptide libraries for five different species of sea anemones from the South China Sea, including *Heteractis crispa*, *Stichodactyla haddoni*, *Exaiptasia diaphana*, *Macrodactyla doreensis*, and *Heteractis magnifica* [3,4,5]. Through multi-omics strategies, we further conducted preliminary pharmacological screening of crude venoms from those sea anemones and found that their venoms exhibited diverse biological activities, including antitumor, analgesic, hypoglycemic, and insecticidal effects [6]. Notably, crude venom from *M. doreensis* showed particularly potent anti-glioma activity, yet this species remains comparatively unexplored.

Glioblastoma is the most common primary malignant brain tumor, characterized by extensive heterogeneity, aggressive invasiveness, and high recurrence [7]. Despite incremental advances in clinical management, outcomes remain poor (5-year survival rate ~7.2%; median overall survival ~21 months), and the disease imposes substantial emotional and economic burdens on families and society [8]. Current therapies remain insufficient due to limited efficacy and the rapid acquisition of drug resistance, underscoring the urgent need for novel therapeutic agents with distinct mechanisms of action [9]. Recent studies have demonstrated that sea anemone–derived peptides display potent antitumor activities across multiple cancer types, including hepatocellular carcinoma, cervical cancer, breast cancer, and ovarian cancer [10,11]. However, their effects on glioblastoma, particularly on invasion-related phenotypes and underlying molecular mechanisms, remain largely unexplored. Venom-derived peptides are a promising class of emerging anticancer agents because they can suppress tumor growth by perturbing cell cycle progression, inhibiting proliferation, and disrupting apoptotic signaling pathways. Among these mechanisms, the tumor suppressor p53 plays a pivotal role as a central transcription factor, orchestrating cell-cycle arrest, senescence, DNA repair, and apoptosis in response to cellular stress and genomic instability. Activated p53 halts the proliferation of mutated or damaged cells and transduces pro-apoptotic signals to ensure effective elimination of aberrant cells, thereby preventing tumorigenesis [12]. Owing to its central regulatory functions, p53 has emerged as a promising therapeutic target across various cancer types.

In this study, crude venom extracted from *M. doreensis* was systematically evaluated through in vitro cellular assays, proteomic profiling, and qPCR validation to elucidate its inhibitory effects on glioblastoma cells and the underlying molecular mechanisms. This work provides a scientific foundation for further exploring the anti-glioma potential of *M. doreensis* venom and contributes to the identification of medically valuable marine-derived peptides as leads for therapeutic development.

## 2. Results

### 2.1. Multi-Omics Identification of M. doreensis Peptides and Network-Pharmacology-Based Screening of Anti-Glioblastoma Targets

**Figure 1:**
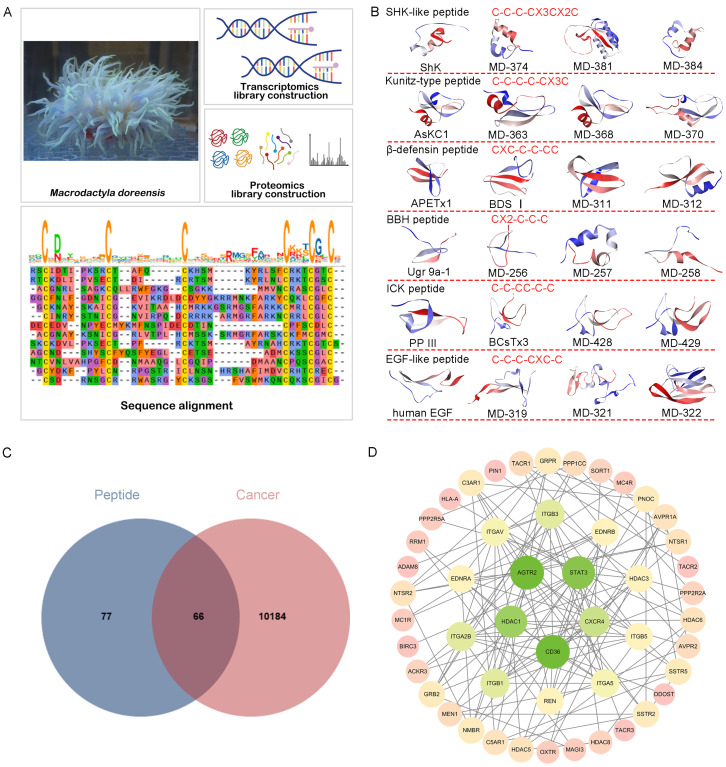
Integrated multi-omics workflow and identification of key peptide families from *M. doreensis*, along with network-pharmacology-based prediction of their anti-glioblastoma potential. (**A**) Schematic overview of the transcriptomics and proteomics workflow, including transcriptome assembly, proteome library construction, and ShK family sequences identified from the transcriptome of *M. doreensis*. (**B**) Sixteen peptides screened from the transcriptome of *M. doreensis* across six different families were subjected to three-dimensional homology modeling. (**C**) Venn diagram of potential therapeutic targets of peptides in the treatment of glioblastoma. (**D**) Target-disease protein–protein interaction (PPI) network ranked by degree centrality.

The sea anemone *M. doreensis* is a common species inhabiting the South China Sea. In our previous work, a peptide library was constructed by integrating transcriptomic and proteomic approaches, yielding 606 sequence reads, including 40 putative peptides from representative families (Figure 1A). These peptides were classified into eight major families according to their cysteine frameworks. Six of these families, including ShK domain, Kunitz-type, β-defensins, boundless β-hairpin (BBH), inhibitor cystine knot (ICK), and EGF-like peptides [13], were prioritized. Sixteen representative peptides (e.g., MD-322, MD-363, MD-374, MD-381, MD-384, and MD-429) were chosen for subsequent structural modeling and functional prediction (Figure 1B).

To identify potential therapeutic targets of peptides derived from *M. doreensis* in glioblastoma, 143 putative targets were predicted using the SEA database [14], while 10,250 glioblastoma-related disease targets were retrieved from GeneCards and OMIM databases [15]. Intersection analysis of these datasets (Figure 1C) resulted in 66 overlapping targets regarded as potential peptide-associated therapeutic targets. These 66 targets were submitted to the STRING database (https://string-db.org/, accessed on 28 November 2025) to construct a protein–protein interaction (PPI) network, comprising 243 nodes and 1208 edges with an average degree of 9.94. Topology analysis performed using Cytoscape [16] identified core nodes based on degree centrality (Figure 1D), with node degree positively correlated with node size and color. Several hub proteins were located at the center of the PPI network, including AGTR2 (angiotensin II receptor type 2), STAT3 (signal transducer and activator of transcription 3), CD36, HDAC1 (histone deacetylase 1), and CXCR4 (C-X-C motif chemokine receptor 4), which have all been reported to participate in glioblastoma-associated signaling networks [17,18,19,20,21]. Collectively, these targets may represent potential therapeutic nodes underlying the anti-glioblastoma activity of *M. doreensis* peptides.

### 2.2. Crude Venom Preferentially Suppresses Glioblastoma Cell Viability

**Figure 2:**
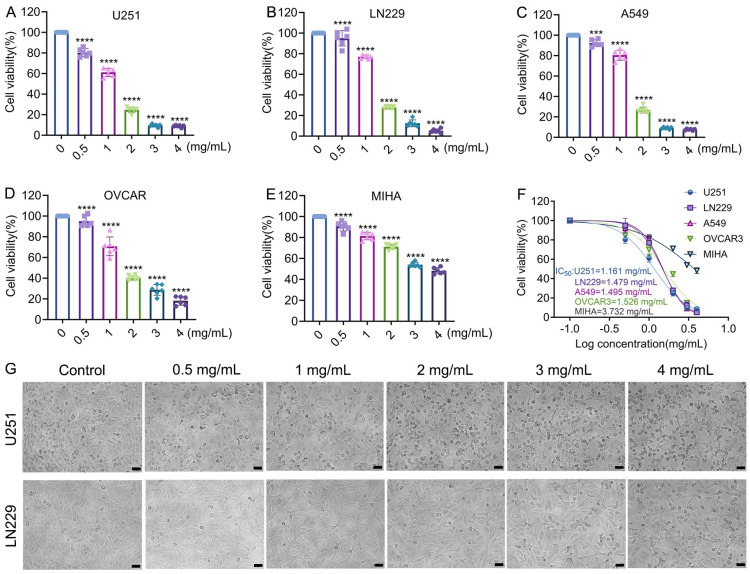
Sea anemone *M. doreensis* crude venom induces dose-dependent cytotoxicity in five cell lines. (**A**–**E**) Cell viability of U251 (**A**), LN229 (**B**), A549 (**C**), OVCAR3 (**D**), and MIHA (**E**) cells following treatment with crude venom at the indicated concentrations (0–4 mg/mL). Viability is expressed as a percentage of the untreated control. Data represent mean  ±  SEM from at least three independent experiments. *** *p* < 0.001, **** *p* < 0.0001 versus control group. (**F**) Dose–response curves generated by nonlinear regression of viability data for U251, LN229, A549, OVCAR3, and MIHA cells. The calculated IC_50_ values are shown. (**G**) Representative bright field (BF) micrographs of U251 and LN229 cells without treatment (control) or treated with different doses of crude venom for 24 h. Scale bars: 100 µm.

In an initial cross-cancer comparison (U251, LN229, A549, OVCAR3, and MIHA), crude venom exhibited the strongest inhibitory effect on glioblastoma cells. Subsequent experiments therefore focused specifically on glioblastoma models U251 and LN229. CCK-8 assays demonstrated significant, dose-dependent cytotoxicity of crude venom in both cell lines (Figure 2A–F). The results showed that although the crude venom also exerted a significant effect on normal MIHA hepatocytes, its cytotoxicity toward U251 cells was markedly higher, with nearly a threefold difference in IC_50_ values (1.161 mg/mL vs. 3.732 mg/mL). Notably, at 3 mg/mL, growth inhibition reached 90.42% in U251 cells and 88.8% in LN229 cells. Consistent with this, IC_50_ values at 24 h were 1.161 mg/mL (U251) and 1.479 mg/mL (LN229) (Figure 2F), indicating a stronger cytotoxic response in U251 cells. This inhibitory effect was further supported by Bright-field (BF) imaging, which further revealed pronounced venom-induced morphological changes in both glioblastoma cell lines (Figure 2G).

To select working concentrations for mechanistic assays while avoiding excessive nonspecific cytotoxicity, we assessed cell viability across a range of doses. At 800 µg/mL, the viability of both U251 and LN229 cells remained within the 60–70% range [22]. Therefore, 800 µg/mL was selected as the highest working concentration for subsequent experiments.

### 2.3. Crude Venom Inhibits Glioblastoma Migration and Invasion

**Figure 3:**
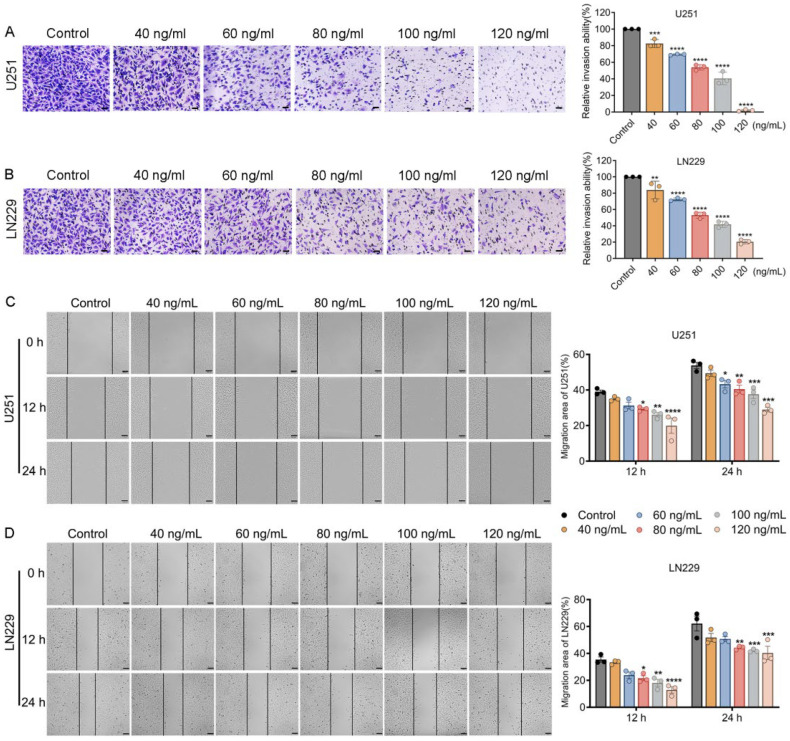
Crude venom suppresses glioblastoma cell migration and invasion in U251 and LN229 cells. (**A**,**B**) Transwell invasion assays showing representative images of invaded U251 (**A**) and LN229 (**B**) cells following treatment with crude venom (40–120 ng/mL). Invaded cells were fixed, counterstained with crystal violet (purple), and imaged. Bar graphs (right) show quantification of relative invasion ability (%) compared with the untreated control. Scale bars: 50 µm. (**C**,**D**) Wound-healing (scratch) migration assays for U251 (**C**) and LN229 (**D**) cells treated with crude venom (40–120 ng/mL). Representative images were captured at 0 h, 12 h, and 24 h, illustrating delayed wound closure with increasing venom concentration. Bar graphs (right) show the quantified migration area (%) at 12 h and 24 h. Scale bars: 100 µm. Data represent mean  ±  SEM from at least three independent experiments. * *p* < 0.05, ** *p* < 0.01, *** *p* < 0.001, **** *p* < 0.0001 versus control group.

Transwell invasion assays showed that crude venom treatment significantly reduced invasion in both U251 and LN229 cells at all tested concentrations compared with the control group without treatment (Figure 3A,B). Notably, U251 cells exhibited greater sensitivity at the highest concentration (120 ng/mL), with invasion reduced to 3.03% versus 20.46% in LN229 cells (Figure 3B), indicating dose-dependent responses and overall dose-dependent suppression of invasion.

Wound-healing assays further demonstrated that untreated controls exhibited substantial gap closure (~40% at 12 h and >50% at 24 h), whereas venom treatment markedly impaired wound closure in both cell lines in a dose-dependent manner, particularly at concentrations ≥80 ng/mL (Figure 3C,D). In line with the invasion data, crude venom exerts a more pronounced inhibitory effect on the migratory capacity of U251 cells compared with LN229 cells. At 24 h, 60 ng/mL significantly slowed down wound closure in U251 cells, while showing no significant effects on LN229 cells. When treated with 120 ng/mL crude venom, U251 cells exhibited a stronger reduction in migration than LN229 cells (28.95% vs. 40.34%).

### 2.4. Crude Venom Suppresses Clonogenic Growth

**Figure 4:**
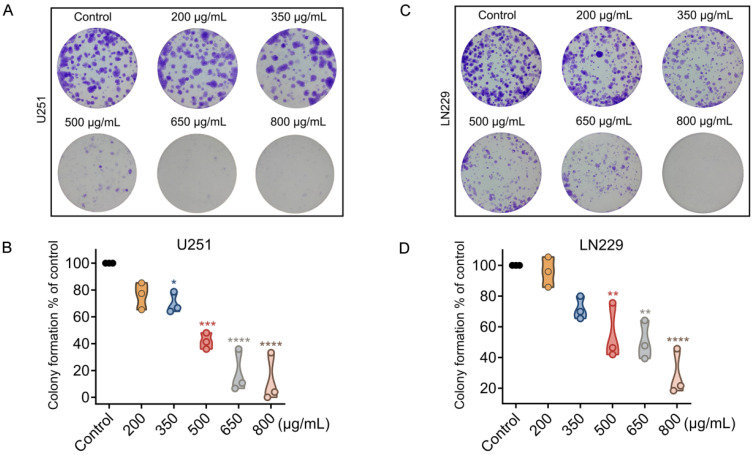
Crude venom inhibits clonogenic survival of U251 and LN229 glioblastoma cells. (**A**,**C**) Representative images of crystal violet–stained colony formation assays performed in U251 (**A**) and LN229 (**C**) cells following treatment with crude venom at indicated concentrations (200–800 µg/mL). (**B**,**D**) Quantification of clonogenic growth for U251 (**B**) and LN229 (**D**) cells, expressed as colony formation (% of control). Violin plots depict the distribution of replicate values. Data represent mean  ±  SEM from at least three independent experiments. * *p* < 0.05, ** *p* < 0.01, *** *p* < 0.001, **** *p* < 0.0001 versus control group.

To evaluate the long-term effects of crude venom on the proliferation of glioblastoma cells, a 21-day colony formation assay was performed [23]. Cell colony counting revealed a significant, dose-dependent inhibition of cell proliferation in crude venom-treated U251 and LN229 cells. Colony formation was nearly abolished by the crude venom at 650 µg/mL in U251 cells and at 800 µg/mL in LN229 cells (Figure 4).

### 2.5. Crude Venom Induces Cell Cycle Arrest in Human Glioblastoma Cells

**Figure 5:**
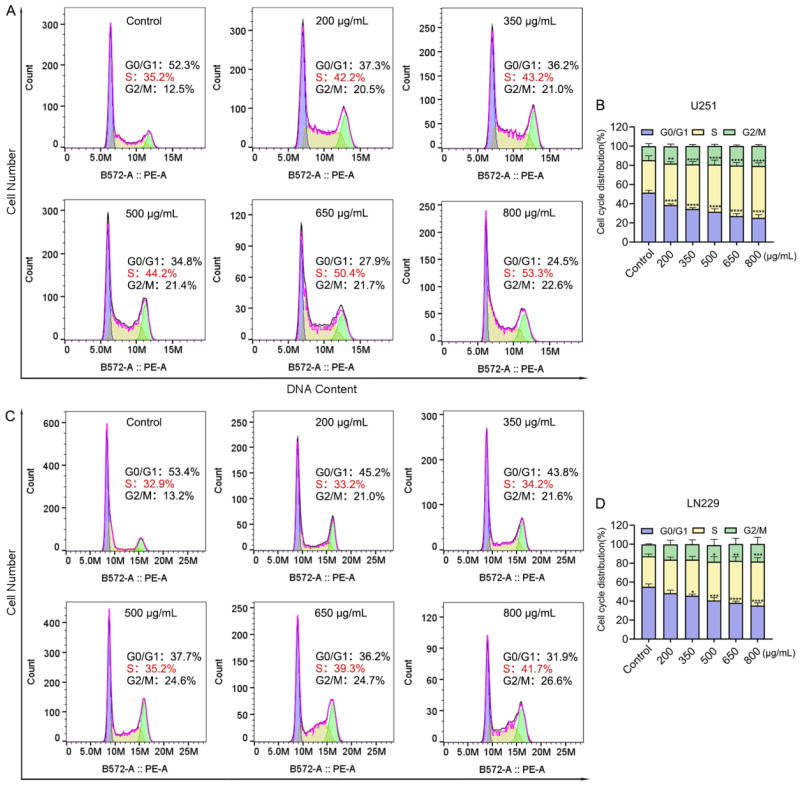
Crude venom induces S-phase accumulation and alters cell cycle distribution in glioblastoma cells. (**A**,**C**) Representative flow-cytometry histograms of DNA content for U251 (**A**) and LN229 (**C**) cells after treatment with different concentrations of crude venom for 24 h. (**B**,**D**) Quantification of U251 (**B**) and LN229 (**D**) cell cycle distribution following treatment with different crude venom concentrations is shown as stacked columns. Data represent mean  ±  SEM from at least three independent experiments. * *p* < 0.05, ** *p* < 0.01, *** *p* < 0.001, **** *p* < 0.0001 versus control group.

To further investigate whether growth suppression involved cell-cycle disruption in glioblastoma cells, the distribution of cells across different phases (G0/G1, S, and G2/M) was evaluated by flow cytometric analysis following PI staining [24]. Consistent with the antiproliferative results, cell cycle analysis revealed a significant increase in the proportion of cells in the S phase upon treatment, suggesting that cell cycle progression may be disrupted (Figure 5). In U251 cells treated with 800 µg/mL crude venom for 24 h, the percentage of cells in the S phase increased significantly from 35.2% in the control group to 53.3%, indicating the S phase arrest (Figure 5A). Meanwhile, the proportion of cells in the G0/G1 phase decreased significantly from 52.3% to 24.5% (Figure 5B). Similarly, 800 µg/mL crude venom treatment for 24 h in LN229 cells led to a significant rise in the S phase population, increasing from 32.9% to 41.7% (Figure 5C), while the proportion of cells in the G0/G1 phase significantly decreased from 53.4% in the control group to 31.9% (Figure 5D).

### 2.6. Crude Venom Induces Apoptosis in Human Glioblastoma Cells

**Figure 6:**
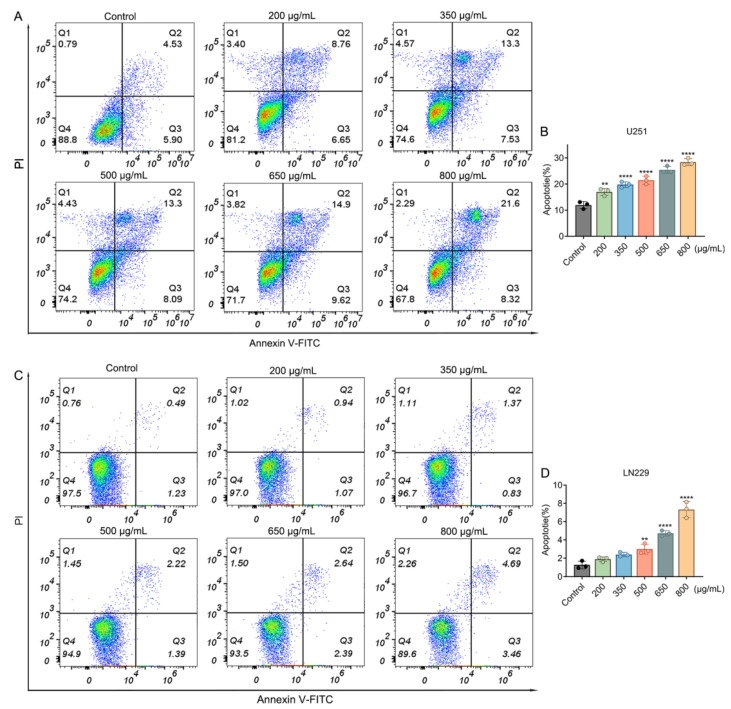
Crude venom induces dose-dependent apoptosis in glioblastoma cells. (**A**,**C**) Representative Annexin V–FITC/propidium iodide (PI) flow-cytometry plots of U251 (**A**) and LN229 (**C**) cells treated with crude venom for 24 h. Quadrants indicate viable cells (Annexin V^−^/PI^−^), early apoptotic cells (Annexin V^+^/PI^−^), late apoptotic/secondary necrotic cells (Annexin V^+^/PI^+^), and primary necrotic cells (Annexin V^−^/PI^+^). Percentages of cells in each quadrant are shown. (**B**,**D**) Quantification of total apoptotic cells (early + late apoptosis; Annexin V^+^) in U251 (**B**) and LN229 (**D**) expressed as a percentage of total cells. Data represent mean  ±  SEM from at least three independent experiments. ** *p* < 0.01, **** *p* < 0.0001 versus control group.

To evaluate whether crude venom induces apoptosis in U251 and LN229 cells, we employed the Annexin V and PI double staining, a method capable of identifying and quantifying apoptotic cells by detecting the binding of cell surface specific markers [25]. The apoptosis rates of U251 and LN229 cells in the control groups were 11.92% and 1.72%, respectively. After 24 h of crude venom exposure, U251 and LN229 cells treated with 800 µg/mL displayed an apoptosis rate of 28.35% and 7.31%, respectively, indicating significant apoptosis induction compared to the control (Figure 6). Although the proportion of apoptotic cells increased with rising concentrations of crude venom, the percentage of necrotic cells labeled as Annexin V^−^/PI^+^ did not show significant changes, suggesting that crude venom primarily exerts its effects by inducing cell apoptosis rather than necrotic cell death.

### 2.7. Proteomics Reveals the Potential Anti-Glioblastoma Targets of Crude Venom

**Figure 7:**
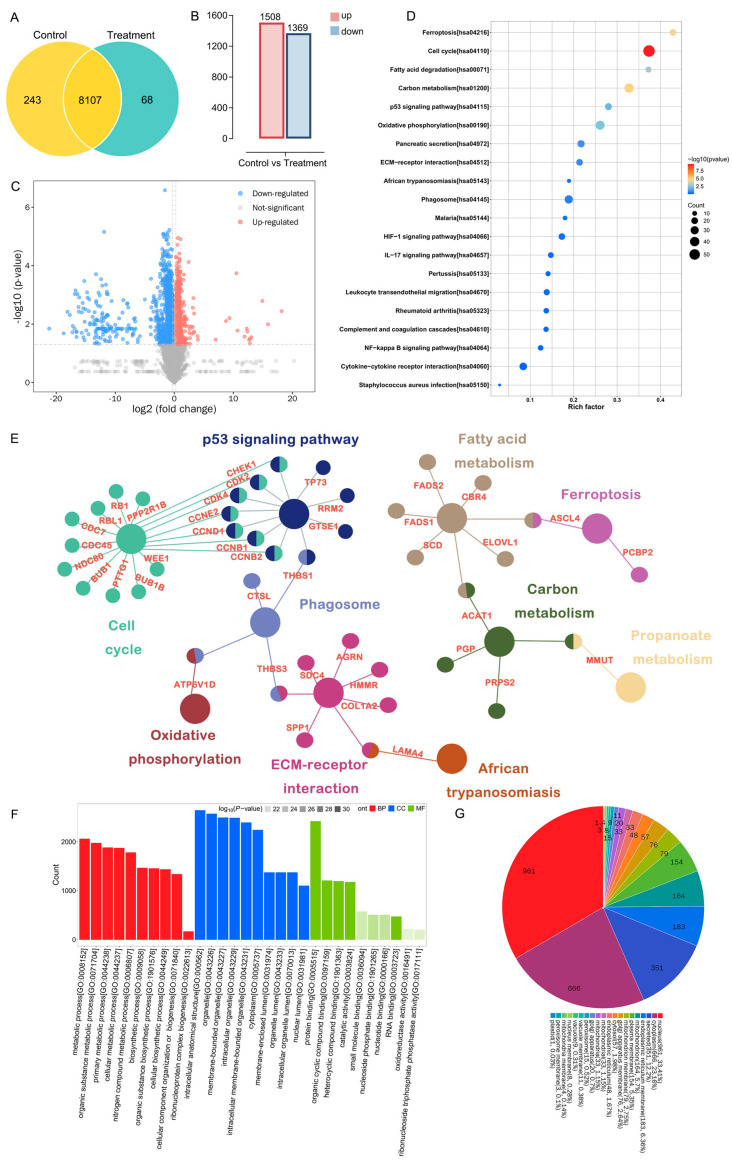
Proteomics revealed the underlying mechanism of crude venom against glioblastoma. (**A**) Venn diagram of differentially expressed proteins. (**B**) Screening plot of differentially expressed proteins. (**C**) Volcano plot of quantitative proteomic analysis, red represents up-regulated proteins and blue represents down-regulated proteins using the threshold of *p* value < 0.05 and Fold change ≤ 0.83 or Fold change ≥ 1.2^2^. (**D**) Top 20 significant KEGG pathways. Dot size represents the number of differentially expressed proteins mapped to each pathway (Count), dot color indicates significance, and the *x*-axis shows the rich factor (ratio of differentially expressed proteins to total annotated proteins in the pathway). (**E**) Gene–pathway network plot linking enriched KEGG pathways to contributing differentially expressed proteins (displayed as gene symbols), highlighting major functional modules including p53 signaling, cell cycle, oxidative phosphorylation, phagosome, ECM–receptor interaction, fatty acid/carbon metabolism, and ferroptosis. (**F**) Gene Ontology (GO) enrichment analysis of differentially expressed proteins across biological process (BP), cellular component (CC), and molecular function (MF) categories. Bar height indicates gene count; color scale reflects significance. (**G**) GO functional classification pie chart showing the subcellular distribution of differentially expressed proteins across enriched GO terms/categories.

To gain deeper insights into the underlying molecular mechanism of crude venom’s action on glioblastoma, quantitative proteomic analysis [26] was performed on control and crude venom-treated U251 cells detecting a total of 8418 expressed proteins (Figure 7A). Using the threshold of *P*_fdr_ < 0.05 and Fold change ≤ 0.83 or Fold change ≥ 1.2^2^, a total of 2877 differentially expressed proteins were identified, including 1508 up-regulated proteins and 1369 down-regulated proteins (Figure 7B,C). Subsequently, Gene Ontology (GO) and Kyoto Encyclopedia of Genes and Genomes (KEGG) enrichment analyses were conducted on the 2877 differentially expressed proteins to explore the potential molecular mechanisms of crude venom in glioblastoma. KEGG enrichment analysis further highlighted significant association with the p53 signaling pathway and cell-cycle regulation, indicating that these pathways may serve as core regulatory mechanisms mediating the venom-mediated antitumor effects (Figure 7D,E). The potential biological functional effects of crude venom on glioblastoma cells were ranked in descending order based on the −log10(*p*-value) corresponding to each entry. GO enrichment indicated prominent changes in metabolic processes, organic substance metabolic process, primary metabolic processes, and cellular metabolic processes (Figure 7F). In terms of cellular components, the proteins were mainly localized to intracellular anatomical structures and organelles. Regarding molecular functions, they were predominantly associated with protein binding, organic cyclic compound binding, and catalytic activity. The subcellular structure annotation and classification revealed that the differential proteins were predominantly localized in the nucleus (33.41%), cytoplasm (23.16%), secreted proteins (12.2%), and endoplasmic reticulum membrane (6.36%), among others (Figure 7G). Together, GO functional classification and subcellular analyses indicated that crude venom inhibits glioblastoma cells primarily by affecting mitochondrial function and metabolic pathways.

### 2.8. Quantitative PCR Validation and Docking Nominate RRM2, CDK2, and CHEK1 as Candidate Targets

**Figure 8:**
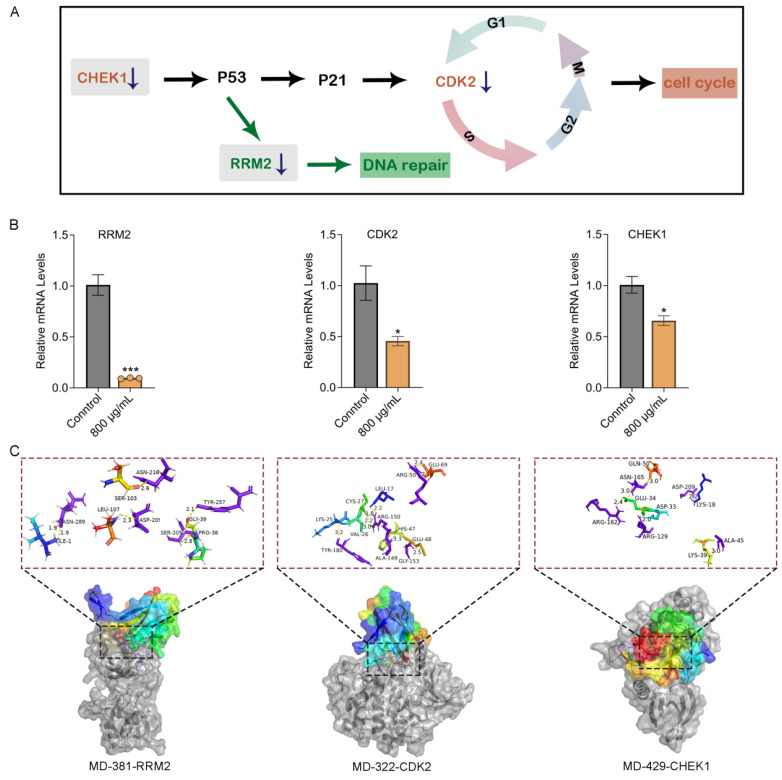
Antiglioblastoma mechanisms of *M. doreensis* crude venom and molecular docking-based validation of its active anticancer targets. (**A**) Schematic model illustrating the proposed mechanism whereby *M. doreensis* venom-induced inhibition/downregulation of CHEK1 activates the p53–p21 checkpoint response, leading to reduced CDK2 activity and cell-cycle arrest, alongside suppression of RRM2 and impaired DNA repair. Downward arrows indicate decreased expression/activity observed after treatment. (**B**) qPCR validation of RRM2, CDK2, and CHEK1 mRNA levels in cells treated with crude venom (800 μg/mL) relative to control. Data were analyzed using an unpaired *t*-test and are presented as mean ± SEM. Statistical significance versus control is indicated (* *p* < 0.05, *** *p* < 0.001). **(C)** Representative 3D molecular docking models showing the interactions of MD-381 with RRM2 (PDB: 7EVR), MD-322 with CDK2 (PDB: 3TI1), and MD-429 with CHEK1 (PDB: 6FCK). Protein surfaces are shown with the predicted binding pockets highlighted; enlarged insets depict key amino acid interactions (e.g., hydrogen bonds and hydrophobic contacts) between each peptide and its target protein. Peptides are colored rainbow and their target proteins are colored purple.

p53 is widely recognized as a central member of the transcription factor family and plays a pivotal role in tumor suppression by regulating cell-cycle arrest, apoptosis, DNA repair, cellular senescence, and differentiation [27]. To identify the key molecular targets potentially regulated by the crude venom, differential protein expression analysis was integrated with KEGG pathway enrichment, GO annotation, and PPI network analysis. Three significantly downregulated genes, RRM2, CDK2, and CHEK1, were identified as central regulatory nodes closely associated with the p53 signaling pathway and cell-cycle control (Figure 8A). RT-qPCR analysis further confirmed that treatment with 800 µg/mL crude venom markedly reduced the expression of RRM2, CDK2, and CHEK1, with fold-change values of 0.09, 0.46, and 0.66, respectively (Figure 8B).

In multi-omics assays, sixteen candidate peptides were predicted from the *M. doreensis* peptide library (Figure 1). To investigate the potential interactions between candidate peptides and these regulatory proteins and thereby screening for peptides that may possess potentially superior biological activity, three-dimensional structural models of these peptides were generated using AlphaFold2, followed by large-scale molecular docking analysis performed in Discovery Studio to evaluate their potential anticancer targets. Several peptides were predicted to form the most stable peptide-protein complexes with the key regulatory targets RRM2, CDK2, and CHEK1. Molecular docking analysis revealed that MD-381 forms sixteen hydrogen bonds and one salt bridge with RRM2, involving residues located within functionally relevant regions, including Pro38–Ser205, Leu107–Asp209, Ser103–Asn216, Gly39–Tyr257, and Ile1–Asn289, consistent with a stable binding mode [28]. In particular, the salt bridge formed between MD-381 and the Gly39-Tyr257 residue pair of RRM2 may contribute to enhanced binding stability and potential interference with RRM2 functional regions. The contact surface areas of the ligand and receptor were 867.44 and 852.98, respectively. Compared with the molecular docking results of other peptides, MD-381 may exhibit more pronounced inhibitory activity toward RRM2. Based on predictive analyses, MD-322 established a total of 26 favorable non-covalent interactions with CDK2 [29], including 19 hydrogen bonds. The contact surface areas between the ligand and the receptor were 777.79 Å^2^ and 755.76 Å^2^, respectively. Notably, MD-322 interacted with residues within the C-(PSTAIRE) helix (e.g., Glu69-Arg50) and those adjacent to the T-loop (Arg150, Arg50), suggesting that the peptide may exert its inhibitory effects by modulating T-loop activation or inducing conformational changes through peptide-protein interactions, rather than directly competing for the ATP-binding hinge region. Previous studies have shown that residues including Arg129 and Arg162 are located within phosphorylation-associated regulatory regions of CHEK1 and contribute to ligand binding [30]. MD-429 was predicted to bind CHEK1 through multiple hydrogen bonds involving residues implicated in ligand recognition and regulatory regions (e.g., Arg129 and Arg162). The contact surface areas between the ligand and the receptor were 913.55 Å^2^ and 932.87 Å^2^, respectively, supporting a plausible mechanism for functional interference (Figure 8C).

## 3. Discussion

Cancer remains one of the most critical global health challenges, with its high incidence and mortality rates continuing to impose a substantial burden on public healthcare systems and patients [31]. Although conventional therapeutic approaches such as surgery, radiotherapy, and chemotherapy have achieved considerable progress, their severe side effects, acquired drug resistance [32], and limited efficacy in advanced-stage cancers continue to hinder clinical outcomes [33]. These unmet medical needs have driven the continuous search for novel, safe, and effective anticancer agents. In this context, natural products, particularly those derived from marine ecosystems, have emerged as a significant reservoir for drug discovery. The extreme and diverse physicochemical conditions of the marine environment have driven marine organisms to evolve unique biosynthetic pathways [34], leading to the production of structurally diverse and biologically potent natural compounds. Within this landscape, peptide toxins isolated from cnidarians (such as sea anemones, jellyfish, and corals) are of particular interest because they often combine high target specificity with strong bioactivity and can modulate cancer-relevant signaling pathways [35]. As a result, they are emerging as highly promising anticancer candidates and may offer new avenues for overcoming the limitations of conventional therapies.

Glioblastoma remains difficult to treat, and standard chemotherapeutic agents, including cisplatin, lomustine, and temozolomide (TMZ) [36,37], are limited by intrinsic and acquired resistance [38]. Therefore, the development of novel, naturally derived compounds with alternative mechanisms of action is highly desired. TMZ, the frontline chemotherapeutic agent for glioblastoma [39], exerts its cytotoxicity primarily through DNA methylation, which activates the mismatch repair pathway and ultimately induces apoptosis. In addition, TMZ typically causes G2/M-phase arrest [40], reflecting its interference with DNA replication fidelity and checkpoint activation. In contrast, the *M. doreensis* crude venom elicited a distinct phenotype, including significant suppression of cell viability, strong suppression of migration/invasion, robust apoptosis induction, and preferential accumulation in S phase, consistent with disruption of DNA synthesis or replication-associated processes rather than the G2/M blockade commonly triggered by TMZ. Lomustine (CCNU), a nitrosourea compound [41], induces cytotoxicity through interstrand crosslinks and protein carbamoylation, resulting in apoptosis and cell-cycle arrest. Although both CCNU and the crude venom promote apoptosis, the apoptotic patterns differ. CCNU typically increases early apoptosis accompanied by prominent DNA fragmentation. In contrast, crude venom treatment led to a pronounced accumulation of late apoptotic cells, suggesting a more sustained or progressive form of cellular stress, potentially mediated by peptide-membrane interactions or modulation of intracellular signaling pathways. Furthermore, CCNU is known to inhibit migration and invasion by suppressing tumor cell adhesion and extracellular matrix (ECM) remodeling. Our wound-healing and Transwell assays demonstrated that the crude venom exerted similar inhibitory effects on glioblastoma cell motility, implying that venom-derived peptides may interfere with cytoskeletal organization or motility-related signaling pathways (Figure 3). Cisplatin exerts its antitumor activity primarily by forming DNA adducts and interstrand crosslinks, which subsequently activate p53-dependent apoptotic pathways [42]. However, cisplatin resistance commonly arises through enhanced DNA repair and impaired apoptotic signaling [43]. In contrast, apoptosis induced by the *M. doreensis* crude venom appears to be less reliant on DNA crosslinking-mediated mechanisms, as evidenced by the observed morphological alterations, elevated cell death as well as the induction of both early and late apoptosis (Figure 2, Figure 4 and Figure 6). These phenotypes suggest involvement of alternative mechanisms, potentially including ion channel modulation, membrane disruption, or peptide-mediated interference with intracellular signaling cascades [44,45,46]. Taken together, these differences suggest that sea anemone-derived peptides may complement existing therapies by engaging distinct mechanisms and targeting alternative vulnerabilities in glioblastoma.

Previous studies have shown that numerous sea anemone toxins, including neurotoxins, actinoporins, pore-forming proteins, and cysteine-rich peptides, can inhibit tumor proliferation, induce apoptosis, or enhance chemosensitivity. For example, APETx4 from *Anthopleura elegantissima* selectively inhibits the oncogenic potassium channel Kv10.1 [38], thereby suppressing proliferation and inducing apoptosis across various tumor types. Similarly, the recombinant actinoporin rHct-S3 from *Heteractis crispa* inhibits the growth of breast, colorectal, and melanoma cancer cells, reduces migration via MMP-2/-9 downregulation [47], and induces apoptosis through caspase activation. Other sea anemone derived toxins, such as ATXII, FraC, EqTx-II, and StI-based immunotoxins [48,49,50], have demonstrated potent cytotoxic effects and potential synergistic actions in anticancer therapy through mechanisms including modulation of sodium and potassium channels, pore formation, membrane disruption, PI3K/Akt pathway inhibition, and mitochondria or caspase dependent apoptosis [51,52,53].

Quantitative proteomics and pathway enrichment implicate p53 signaling and cell-cycle control as central axes underlying the anti-glioblastoma effects of *M. doreensis* crude venom. We further identified three downregulated genes, RRM2, CDK2, and CHEK1, and validated their suppression by qPCR (Figure 7B). These targets are mechanistically consistent with S-phase perturbation and checkpoint dysregulation and are frequently implicated in glioblastoma proliferation and therapeutic resistance. Moreover, to improve component-level resolution, we integrated multi-omics resource development, functional screening, and mechanistic inference and identified three candidate peptides, MD-381, MD-322, and MD-429, from *M. doreensis* as candidates capable of engaging these targets. The interactions between these peptide toxins and the target proteins were structurally evaluated using 3D modeling and molecular docking (Figure 7C).

Interestingly, crude venom appears to exert anticancer activity through multiple pathways, rather than through a single classical mechanism such as membrane disruption or ion-channel modulation. MD-381, an ShK domain peptide resembling traditional sea-anemone potassium channel blockers, formed a highly stable complex with RRM2, which is an essential regulator of DNA synthesis and a major driver of glioblastoma progression. MD-322, an EGF peptide, exhibited strong binding to CDK2, a central kinase controlling DNA replication and S-phase progression. MD-429, an ICK peptide, demonstrated high affinity for CHEK1, a key checkpoint kinase in the DNA-damage response. Therefore, our study expands the understanding of sea anemone toxins from their classical roles in ion channel modulation to intracellular signaling targets, thereby providing an effective strategy for discovering novel anticancer agents from marine organisms.

In future studies, specific peptides will be screened from multi-omics databases, followed by solid-phase peptide synthesis (SPPS) and structural identification. Their anticancer activities will then be systematically evaluated through in vitro and in vivo experiments. However, despite the strong therapeutic potential demonstrated by sea anemone-derived peptides, their application in glioblastoma treatment remains constrained by several inherent challenges, most notably the limited ability of peptide molecules to penetrate the blood–brain barrier (BBB). Therefore, encapsulating the screened candidate peptides into nanodelivery systems [54] or combining them with targeted drug delivery systems such as peptide-drug conjugates (PDCs) [55] may help enhance their BBB penetration and tumor targeting. Such targeted delivery strategies may provide a feasible approach for exploring peptide-based anti-glioblastoma therapies and for overcoming the limitations of conventional chemotherapy.

## 4. Materials and Methods

### 4.1. Cell Lines

Human glioblastoma cell lines (U251 and LN229), lung carcinoma cells (A549), ovarian cancer cells (OVCAR3), and normal human hepatocyte cell line (MIHA) were obtained from the Key Laboratory of Carcinogenesis and Intervention of Hainan Province. U251, LN229, and A549 cells were cultured in DMEM supplemented with 10% fetal bovine serum (FBS) at 37 °C in a humidified atmosphere containing 5% CO_2_. OVCAR3 and MIHA cells were maintained in 1640 medium containing 10% FBS under the same culture conditions.

### 4.2. Animals and Venom Extraction

Specimens of *M. doreensis* used in this study were collected from the South China Sea (18°N, 112°E) at a depth of approximately 4 m in June 2024. The collected sea anemones were maintained in glass aquariums containing filtered natural seawater under controlled laboratory conditions for 1–2 weeks to allow acclimatization. Crude venom extracts were subsequently obtained from fresh tissues using a homogenization method as previously described [3].

### 4.3. Identification of Overlapping Targets Between Sea Anemone Peptides and Glioblastoma and Construction of the Protein–Protein Interaction Network

The peptide sequences were first imported into ChemDraw (version 23.1.1) to obtain their SMILES IDs, which were then submitted to the SEA database (https://sea.bkslab.org/, accessed on 19 November 2025) to identify potential peptide-related targets. Subsequently, the term “glioblastoma” was used as a search keyword to retrieve glioblastoma-associated disease targets from the GeneCards (https://www.genecards.org/, accessed on 23 November 2025) and OMIM (https://omim.org/, accessed on 23 November 2025) databases. The results from the two databases were merged, and duplicate targets were removed. A Venn diagram analysis was then performed to illustrate the overlapping targets between glioblastoma and the peptides. Protein–protein interaction (PPI) analysis was conducted to more effectively identify core regulatory genes. The genes identified as overlapping targets between the peptides and the disease were imported into the STRING database. The database parameters were set to focus on the “Homo sapiens” species, with the PPI confidence score threshold set to 0.4. Nodes with interaction scores greater than 0.4 were retained, and those with higher degree values were identified as core targets. The network of potential key targets was constructed using Cytoscape software (version 3.9.1) [56].

### 4.4. Bioinformatics Analysis

To elucidate the functions of the core targets and investigate their associated biological processes and signaling pathways, enrichment analyses were performed on the core targets. Differentially expressed proteins between control and crude venom-treated U251 cells were identified using the limma R package (v4.0). Subsequently, Gene Ontology (GO) enrichment analyses (BP, MF, and CC) and KEGG pathway analyses were conducted, and the results were visualized using bar plots and bubble plots [57].

### 4.5. Cell Viability Assay

U251, LN229, A549, and OVCAR3 cells were seeded into 96-well plates at a density of 1 × 10^4^ cells per well and treated with various concentrations of crude venom (0, 0.5, 1, 2, 3, and 4 mg/mL; and 100 µL per well). After 24 h of incubation, cell viability was assessed using the CCK-8 assay [58]. Briefly, 100 µL of CCK-8 solution was added to each well, followed by a further 2 h incubation at 37 °C. The absorbance at 450 nm was then measured using a microplate reader.

### 4.6. Wound Healing (Scratch) Experiment

The wound-healing assay was performed to evaluate the migratory capacity of U251 and LN229 cells. Log-phase cells were seeded into 6-well plates at a density of 1 × 10^5^ cells per well [59]. After serum starvation for 24 h, a straight scratch was created across the cell monolayer using a sterile 200 µL pipette tip. Cellular debris was removed by washing the wells three times with PBS. The cells were then incubated in serum-free medium containing different concentrations of crude venom at 37 °C in a 5% CO_2_ incubator, while untreated cells served as the control group. Images of the wound area were captured at 0, 12, and 24 h using an inverted microscope (10× magnification). Wound area was quantified using ImageJ 1.8.0 software [60].

Wound closure (%) was calculated as Equation (1):

[(A_0_ − A*_t_*)/A_0_] × 100 (1)

where A_0_ is the initial wound area and A*_t_* is the wound area at time *t*.

### 4.7. Transwell Invasion Assay

Transwell chambers were coated with matrigel (1:8 dilution) at 37 °C for 3 h [61]. U251 and LN229 cells (8 × 10^4^ per well) were then seeded into the upper chamber and treated with crude venom at final concentrations of 40, 60, 80, 100, and 120 ng/mL. Cells without venom treatment served as the control group. DMEM supplemented with 10% FBS was added to the lower chamber as a chemoattractant. After incubation at 37 °C for 24 h, invaded cells were fixed with 4% paraformaldehyde for 30 min, stained with 0.1% crystal violet for 20 min, and subsequently imaged under a light microscope (20× magnification). The number of invading cells was counted using ImageJ.

### 4.8. Colony Formation Assay

A single cell that undergoes continuous proliferation for more than six generations in vitro can form a colony, which serves as an indicator of cellular proliferative capacity and clonogenic potential. U251 and LN229 cells were seeded into 6-well plates at a density of 1000 cells per well. The medium was not replaced during the first week to allow colonies to form under the given culture conditions. After one week, the culture medium was removed, and cells were treated with crude venom at concentrations of 200, 350, 500, 650, and 800 µg/mL, while untreated cells served as the control group. After 24 h of treatment, the medium was replaced with fresh DMEM supplemented with 10% FBS, and cells were cultured for an additional week. At the end of the incubation period, the cells were washed twice with pre-cooled PBS, fixed with 4% paraformaldehyde for 20 min, and stained with 0.1% crystal violet for 20 min. The number of colonies was quantified using ImageJ software.

### 4.9. Cell Cycle Analysis by Propidium Iodide (PI) Staining

Cell-cycle distribution was analyzed by flow cytometry using a DNA content-based quantification method. U251 and LN229 cells were seeded into 6-well plates at a density of 1.5 × 10^5^ cells per well and incubated for 24 h [62], followed by treatment with crude venom at concentrations of 200, 350, 500, 650, and 800 µg/mL. Untreated cells served as the control group. After 24 h of incubation, cells were harvested and fixed in 70% ice-cold ethanol at 4 °C for 12 h. The fixed cells were centrifuged at 1000× *g* for 5 min, and the resulting pellet was collected, resuspended in 1 mL PBS, and centrifuged again to remove residual ethanol. The cell pellets were then treated with RNase A for 30 min and subsequently stained with propidium iodide (PI) for flow cytometric analysis. The proportion of cells in each phase of the cell cycle was quantified using FlowJo_v10.10.0 software.

### 4.10. Apoptosis Quantification by Flow Cytometry

Flow cytometry was used to evaluate cell viability, apoptosis, and necrosis based on staining with specific fluorescent markers. U251 and LN229 cells were seeded into six-well plates at a density of 1.5 × 10^5^ cells per well and allowed to adhere for 24 h. The cells were then treated with crude venom at concentrations of 200, 350, 500, 650, and 800 µg/mL for an additional 24 h [63]. Untreated cells served as the control group. After incubation, cells were harvested using EDTA-free trypsin and centrifuged at 1000× *g* for 5 min. The supernatant was discarded, and the cell pellet was resuspended in 195 µL of 1x Annexin V-FITC binding buffer. Subsequently, 5 µL of Annexin V-FITC and 10 µL of PI were added, gently mixed, and incubated for 10 min at room temperature in the dark. The stained cells were analyzed using a flow cytometer to determine the proportions of viable cells, early apoptotic cells, late apoptotic cells, and necrotic cells.

### 4.11. Quantitative PCR

Total RNA was extracted using RNA extraction reagent (Servicebio, Wuhan, China) according to the manufacturer’s instructions. Real-time quantitative PCR (qPCR) was performed using SweScript All-in-One RT SuperMix for qPCR (Servicebio, China) with gene-specific primer-probe sets on a Bio-Rad fluorescence quantitative PCR system (Bio-Rad, Shanghai, China). Each sample was analyzed in technical triplicate. The sequences of gene-specific primers are listed in Supplementary Table S1. Relative gene expression was normalized to GAPDH [64] and analyzed using CFX Maestro software system.

### 4.12. DIA Proteomics and Bioinformatics

Proteomic analysis was performed using liquid chromatography-mass spectrometry (LC-MS) technology. For each sample, 500 ng of total peptides were separated and analyzed with a nano-UPLC (Vanquish neo) coupled to an Astral instrument (Thermo Fisher Scientific, Shanghai, China) with a nano-electrospray ion source. Data independent acquisition (DIA) was performed in profile and positive mode with an Orbitrap analyzer (Thermo Fisher Scientific, Shanghai, China) at 240K resolution over *m*/*z* 380–980 for MS1. MS2 scans covered *m*/*z* range of 150–2000, with absolute AGC Value 5E4, and maximum injection time 3 ms using HCD with normalized collision energy (NCE) of 25% and an isolation window of 2 *m*/*z*.

Raw MS files were processed using DIA-NN software 2.2.0. MS spectra lists were searched against their species-level UniProt FASTA databases. Carbamidomethyl [C] was set as a fixed modification; oxidation (M) and Protein N-terminal acetylation were variable modifications. Trypsin was used as a protease to break down proteins. A maximum of 2 missed cleavages was allowed. The false discovery rate (FDR) was set to 0.01 for both PSM and peptide levels. Peptide identification was performed with an initial precursor mass deviation of up to 20 ppm and a fragment mass deviation of 20 ppm. All the other parameters were default.

### 4.13. Molecular Docking

To elucidate the molecular interactions and binding modes between representative peptides and key target proteins (Supplementary Table S2), employing the AlphaFold Colab tool (https://colab.research.google.com/github/sokrypton/ColabFold/blob/main/AlphaFold2.ipynb, accessed on 12 September 2025) [65,66]. Energy minimization of the 3D peptide structures was performed using ChemBio 3D (version 14.0.0). The crystal structures of the receptors were obtained from the RCSB Protein Data Bank (http://www.pdb.org/, accessed on 21 October 2025) and subsequently modified using Discovery Studio 2019. The modifications included hydrogen addition, ligand and water removal, amino acid optimization, and charge computation. Docking was performed using ZDOCK (a rigid protein docking algorithm), followed by refinement with RDOCK (evaluating the binding configuration of the complex). Predicted interactions, including hydrogen bonding, π-Alkyl, π-Sigma interactions, and hydrophobic contacts, were analyzed. Lastly, PyMol software 3.1.5.1 facilitated the visualization of the docking results on the optimal conformation.

### 4.14. Statistical Analysis

All experiments were performed in triplicate unless otherwise stated. Data analysis was carried out using GraphPad Prism version 10.1.2; images were processed using Adobe Photoshop 20.0.4 software. Results are presented as mean ± standard deviation (SD) or mean ± standard error of the mean (SEM), as appropriate. Statistical significance was evaluated using an unpaired *t*-test or one-way/two-way ANOVA as appropriate, with a *p*-value ≤ 0.05 considered statistically significant. Statistical significance is denoted as **** (*p* value <0.0001); *** (*p* value <0.001); ** (*p* value <0.01); * (*p* value <0.05); or ns (not significant).

## 5. Conclusions

This study provides integrated evidence supporting the potent anti-glioblastoma activity of the crude venom extracted from *M. doreensis* and identifies promising peptide candidates derived from it. Through a multi-faceted approach combining in vitro functional validation, quantitative proteomics, and computational modeling, we have shown that venom treatment suppresses glioblastoma cell viability, inhibits migration and invasion, induces apoptosis, and promotes S-phase cell-cycle arrest. Mechanistically, proteomic enrichment analyses implicate the p53 signaling network and cell-cycle regulation, and we identified RRM2, CDK2, and CHEK1 as downregulated candidate mediators. Furthermore, we have pinpointed three candidate peptides, MD-381, MD-322, and MD-429, that demonstrate high-affinity predicted binding to the identified pivotal glioblastoma associated targets (RRM2, CDK2, and CHEK1). Collectively, these findings underscore *M. doreensis* venom as a rich and promising source of novel anti-glioma bioactive agents and delineate a clear mechanistic framework centered on cell cycle disruption and p53-mediated apoptosis, supporting future isolation and experimental validation of individual peptides for therapeutic development.

## Data Availability

All datasets generated or analyzed for this study are included in the manuscript and the Supplementary Files.

## Supplementary Materials

The following supporting information can be downloaded at: https://www.mdpi.com/article/10.3390/md24030092/s1, Table S1: Primer sequences, fragment lengths, and annealing temperatures for RRM2, CDK2, and CHEK1; Table S2: Molecular docking parameters of representative peptides from the sea anemone *M. doreensis* with the target proteins CDK2, CHEK1, and RRM2, including the total number of hydrogen bonds, total salt bridges, ligand contact surface area, and receptor contact surface area.
